# Real-World Social Reward Processes are Linked to Momentary Positive Affect in Adolescent Girls

**DOI:** 10.1007/s10802-024-01276-9

**Published:** 2024-12-12

**Authors:** Stefanie L. Sequeira, Julianne M. Griffith, T. H. Stanley Seah, Kiera M. James, Cecile D. Ladouceur, Jennifer S. Silk

**Affiliations:** 1 Department of Psychology, University of Virginia, 485 McCormick Rd, Rm 226K, Charlottesville, VA 22904 USA; 2 Department of Psychiatry, University of Pittsburgh, Pittsburgh, PA USA; 3 Department of Psychology, University of Pittsburgh, Pittsburgh, PA USA

**Keywords:** Adolescence, Social reward, Peers, Positive affect, Ecological momentary assessment

## Abstract

Positive peer interactions are critical for adolescent development and well-being. Showing little interest in interacting socially with peers and/or extracting little reward from positive peer interactions can be markers of social anhedonia, which impacts many youths, especially girls, with social anxiety and depressive disorders. Reduced interest or reward in peer interactions may contribute to social anxiety and depression in girls through effects on positive affect (PA), though associations between social anhedonia and momentary PA have yet to be tested. The present study used ecological momentary assessment to test such associations between real-world anticipatory social reward (i.e., interest in upcoming peer events), consummatory social reward (i.e., reward extracted from positive peer interactions), and momentary PA in a sample of 129 girls (aged 11–13 years) who were oversampled for high shy/fearful temperament, a risk factor for future social anxiety and depression. Girls reported higher PA following a more socially rewarding peer interaction, and higher PA on days they reported higher anticipatory social reward. Exploratory analyses showed that these associations were specific to PA; neither anticipatory nor consummatory social reward was associated with changes in negative affect. Findings may inform the development of clinical interventions that target social anhedonia to modify PA in youth with affective disorders.

Positive social experiences, particularly amongst peers, play a crucial role in shaping development and well-being during adolescence. Positive peer experiences help adolescents navigate complex social contexts to achieve key developmental tasks of joining peer groups and forming close, meaningful relationships (Brown & Larson, 2009). Normative increases in social motivation, likely supported by maturation in social-affective neural circuitry (Crone & Dahl, 2012), and increased time spent with peers in person and online may contribute to more positive peer experiences during adolescence. However, individual differences exist in the extent to which adolescents enjoy or look forward to potentially positive social experiences. Social anhedonia, or reduced interest and pleasure in social interactions and relationships, is a key symptom and correlate of several psychiatric disorders that emerge during adolescence, especially in girls, including depression and social anxiety disorder (Barkus & Badcock, 2019; Richey et al., 2019). Many clinical interventions for affective disorders thus focus on modulating an individual’s interest and reward felt in social interactions, with the goal of increasing positive affect (PA) (e.g., Positive Affect Treatment [Craske et al., 2019]; Engage & Connect [Solomonov, 2023]). Notably, however, within-person, longitudinal associations between real-world social interest, reward, and PA in the context of peer social interactions have yet to be tested empirically in youth. Understanding how social interest and reward are associated with PA in the daily lives of adolescent girls at temperamental risk for social anxiety and depression, the goal of the present study, may inform the development of treatment approaches that target social reward processes in adolescents with affective disorders.

Social interest and pleasure may correspond to anticipatory and consummatory social reward processes, respectively (Aldridge-Waddon et al., 2020; Barkus & Badcock, 2019; Rademacher et al., 2017). Moreover, general reward processing can be distinguished by psychological components related to reward ‘wanting’, reward ‘liking’, and reward learning, which have dissociable neural mechanisms (Berridge & Robinson, 2003; Berridge et al., 2009). Reward ‘wanting’ refers to incentive salience, an anticipatory and motivational process that promotes approach towards potential rewards. Reward ‘liking’ refers to a consummatory process associated with subjective pleasure that individuals extract from rewards. The mesocorticolimbic neural circuitry involved in reward ‘wanting’ and ‘liking’ undergoes substantial maturation during adolescence (Reynolds & Flores, 2021), which may contribute to normative increases in motivation during this developmental period (Van Duijvenvoorde et al., 2016). These neural changes occur in tandem with substantial social changes, such as a shift in focus from the family to the peer environment, increased opportunities to engage socially with peers, and heightened social sensitivity (Blakemore & Mills, 2014). Thus, studying reward ‘wanting’ and ‘liking’ in the context of social interactions and experiences is of central importance during adolescence, especially for girls. Adolescent girls are particularly sensitive to social evaluation (Rudolph & Conley, 2005) and highly value social goals (Rose & Rudolph, 2006).

Higher social reward ‘wanting’ during adolescence may help youth approach new peers to join peer groups and form close relationships, contributing to better mental health and well-being. On the other hand, reduced social approach motivation is a symptom or correlate of several psychiatric disorders and symptoms that affect youth, including depression, social anxiety, and psychosis (Barkus & Badcock, 2019; Martin et al., 2024). In the context of these disorders (and also in typically-developing individuals), reduced social approach motivation or social reward ‘wanting’ are thought to play a role in maintaining and exacerbating low PA (Barkus, 2021; Blanchard et al., 1998; Craske et al., 2019; Kerns et al., 2008). For the current study, we assess potential social reward ‘wanting’ in girls by measuring anticipatory social reward, or the degree to which youth look forward to an upcoming social event that has the potential to be socially rewarding.

Youth higher in social reward ‘liking’ may extract more pleasure or social reward (e.g., connectedness, belongingness) from positive social interactions. Moreover, higher social pleasure or social reward may contribute to better mental health by increasing PA. In support of this hypothesis, Craske and colleagues (2023) recently found that for adults low in PA, an intervention targeting reward hyposensitivity increased reward anticipation-motivation and response to reward attainment, and these increases correlated with improvements in self-reported PA and symptoms of depression and anxiety. This intervention, and others designed for individuals experiencing less pleasure (e.g., individuals with a depressive disorder), teach skills like mindfulness, savoring, and gratitude that help individuals focus on the positive, rewarding aspects of their experiences, which may help upregulate PA. Of note, associations between anticipatory or consummatory social reward processes and PA are likely reciprocal, such that increasing PA may also contribute to higher social reward anticipation-motivation and pleasure (Craske et al., 2019). These associations align with the broaden-and-build theory of positive emotions (Fredrickson, 2001; Garland et al., 2010); experiencing PA may spark the urge to connect socially and enhance one’s capacity for experiencing social reward in their social interactions, which then works to reinforce PA.

Anticipating and experiencing pleasure and reward in social interactions is thus thought to be closely related to adolescent psychopathology through associations with PA. However, within-person associations between anticipatory social reward (i.e., how much youth look forward to potentially positive social experiences), consummatory social reward (i.e., how much reward they extract from a social interaction), and PA have yet to be systematically examined in youth. The advancement of ambulatory assessment methods provides promising opportunities for addressing this important gap in the literature and testing these associations with high ecological validity.

The present study capitalized on such advancements to examine associations between real-world social reward process and PA in 129 early adolescent girls (ages 11–13), two-thirds of whom were recruited to be high in shy and/or fearful temperament as reported by the child and/or their parent to increase variability in social anxiety symptoms. Relative to boys, early adolescent girls are particularly sensitive to social evaluation (Rudolph & Conley, 2005) and at higher risk for many disorders associated with blunted PA, including social anxiety and depression (Merikangas et al., 2010). Thus, early adolescent girls are an important population in which to test associations between social reward processes and PA.

For this study, a 16-day ecological momentary assessment (EMA) protocol was administered to assess girls’ anticipatory social reward each morning, social reward extracted from peer interactions throughout the day, and momentary PA. Multilevel structural equation modeling was used to disaggregate within- and between-persons effects of social reward on momentary PA. In the context of EMA, between-persons variance refers to stable, trait-like differences in a specific variable of interest (e.g., social reward) across sampling occasions, while within-persons variance refers to deviations from one’s own average of a specific variable of interest at any given sampling occasion. Decomposing variance into within- and between-persons components is essential to generating a nuanced understanding of key within-persons processes of interest (above and beyond between-persons variance) (Roesch et al., 2010). Theories of change are often predicated on within-persons assumptions (e.g., intervening on mechanism X will lead to change in outcome Y for a given individual), and an understanding of within-persons mechanisms of change is essential to advancing interventions for mental health concerns. In the context of the present investigation, the disaggregation of effects into between- and within- persons components can provide insight into whether youth higher in social reward than their peers tend to experience higher PA than their peers (a between-persons effect), and also whether incremental increases in an adolescent’s experience of social reward at time t is related to corresponding increases in their momentary experience of positive emotions at time t + 1 (a within-persons effect). Together, these results paint a rich picture of the role of daily-life social reward processes in contributing to individual differences in PA and to intraindividual, dynamic changes in PA.

We hypothesized that at both within- and between-person levels, higher anticipatory social reward (i.e., looking forward to future social event) would be positively associated with higher PA for the rest of the day, while perceiving higher social reward in recent positive peer interactions would be positively associated with higher momentary PA. We also hypothesized that at both within- and between-person levels, higher consummatory social reward over the course of the day would be associated with higher anticipatory social reward the next morning.^1^^,^^2^ Though we focused analyses on PA, sensitivity (not pre-registered) analyses tested associations between social reward and NA to speak to specificity of findings. We anticipated that associations between social reward and affect would be restricted to PA, aligning with recent EMA research in youth ages 12–15 showing that positive social experiences on social media were associated concurrently with higher PA but not NA (Sequeira et al., under review).

## Methods

### Participants

One-hundred-twenty-nine girls ages 11 to 13 were recruited for a longitudinal study on girls’ development via online advertisements and announcements in the community. Girls were recruited based on parent-reported sex at birth and enriched for shy/fearful temperament, which was assessed using the Early Adolescent Temperament Questionnaire- Revised (EATQ-R; Ellis & Rothbart, 2001). Parents completed the EATQ-R (Parent) during screening for the study (which took place via phone or online). Youth participants completed the EATQ-R (Child) during their first visit, which could lead to re-assignment of participants into different temperament groups. The sample was stratified such that approximately 2/3 of participants (*n* = 85) scored > 0.75 SDs above the previously established mean on the parent- or adolescent-rated fear scales (3.12 for parent-report, 3.48 for adolescent-report) or shyness scales (2.99 for parent-report, 3.16 for adolescent-report). All other participants (*n* = 44) scored below this cut-off and were considered to be in the normative range of shy/fearful temperament.

Exclusionary criteria included a current or lifetime DSM-5 diagnosis of any anxiety disorder (except specific phobia), obsessive-compulsive disorder, post-traumatic stress disorder, major depressive disorder, or any psychotic or autism spectrum disorder, as determined by the Kiddie-Schedule for Affective Disorders and Schizophrenia (K-SADS-PL; Kaufman et al., 1997). Additional exclusionary criteria included IQ < 70 as assessed using the Wechsler Abbreviated Scale of Intelligence-Second Edition (WASI-II; Wechsler, 2011), lifetime presence of a neurological or serious medical condition, presence of any MRI contraindications, presence of head injury or congenital neurological anomalies (based on parent report), acute suicidality, and medications that affect the central nervous system. Usable EMA data were available for 117 out of 129 participants. One participant withdrew from the study prior to the EMA protocol, one was unable to participate in EMA due to scheduling conflicts, four completed < 25% of all EMA surveys and were thus excluded, four participants withdrew participation during the EMA protocol, and data from two participants were lost due to technological malfunction. Included participants were primarily white (68.4%) and median family income was between $80,0001–90,000, with a range from less than $20,000 to over $100,000. See Table 1 for basic demographic and clinical information.

**Table 1:** Participant (*N* = 117) demographics & clinical information

	M	SD
**Age**	12.25	0.80
**Race/Ethnicity**	**N**	**%**
Asian/Asian American	2	1.7
Biracial	11	9.4
Black/African American	22	18.8
Native American	1	0.9
Other racial background	1	0.9
White/European American	80	68.4
Latine or Hispanic	10	8.5
Total Family Income (Annual)*		
$0–20,000	10	8.7
$20,001–40,000	9	7.8
$40,001–60,000	16	13.9
$60,001–80,000	18	15.7
$80,001-100,000	16	13.9
$100,001 +	46	40.0
**Diagnoses**		
Specific phobia	18	15.4
Attention-deficit/hyperactivity disorder		
Primarily inattentive type	3	2.6
Combined type	3	2.6
Unspecified	1	0.9
Oppositional defiant disorder	5	4.3
Unspecified disruptive behavior disorder	1	0.9
Adjustment disorder	1	0.9
Enuresis	1	0.9
Tic Disorder	2	1

Note. *Income data are missing for two participating families

### Procedures

The study was approved by the University of Pittsburgh Institutional Review Board. Parents and youth provided informed consent and assent, respectively. Following informed consent, the WASI and K-SADS-PL were administered to determine eligibility. During a follow-up visit to the lab, approximately two weeks after the initial visit, participants completed a cell-phone EMA home protocol. Youth were provided a pre-programmed android smartphone on which they entered responses to a series of questions about their emotions and daily experiences with peers for 16 consecutive days (ten weekdays and six weekend days) using a secure smartphone app for Web Data Express (WDX) developed by the Office of Academic Computing in the University of Pittsburgh Department of Psychiatry. Adolescents were randomly sampled (i.e., received an electronic notification to respond) three times per day on weekdays (once in the morning between 7 AM and 8 AM and twice between 4 PM and 9:30 PM) and four times per day on the weekends between 10 AM and 9:30 PM, allowing for a maximum of 54 observations. Participants completed an average of 81.3% of prompts (SD = 13.9%, range = 37.0 − 100%). Youth participants were compensated for completing the initial interview, which included the KSADS-PL, WASI-II, and questionnaires; parents were also compensated for completing the KSADS-PL and questionnaires. Youth were also compensated (up to $10/day) for completing the EMA protocol, with a bonus (an additional $8-$20) provided for high EMA compliance.

### EMA Measures

A summary of EMA measures analyzed for this study can be found in Table 2.

**Anticipatory social reward.** To assess anticipatory social reward, or anticipation of a positive social experience, girls were asked, “What are you most looking forward to today?”. This question was asked only once daily (during the first morning assessment). Girls typed out details about what they were looking forward to, how much they were looking forward to it (using a 0-100 sliding scale), and with whom the event would be occurring. In primary analyses, we restricted events to only events with peers, given the salience of peer relationships and interactions during adolescence.

**Consummatory social reward.** To assess youths’ perceptions of social reward in their daily peer interactions, girls were asked at each prompt to think about the interaction with peers (i.e., other kids their age) that made them feel the best since the last prompt (see Table 2). Participants were asked to type out details about this interaction and prompted if they reported having difficulty thinking about a recent positive peer interaction. If participants continued to indicate that they did not have a positive peer interaction after being prompted, they were asked to type out details about their last positive interaction with anyone (these responses were not included in present analyses). After typing out details about the interaction, participants were asked to indicate when the interaction occurred and who was involved. For the present study, we included interactions with friend or friend(s), romantic partners, and other kid(s). Participants were then asked to use a check-box to indicate how they felt during the peer interaction. Response options consisted of eight “social reward” items that assess how socially rewarded girls felt in positive interactions with their peers (Table 2). These items were summed for each interaction as an index of perceived social reward in that positive peer interaction.

To evaluate the structure of the eight-item scale and establish preliminary reliability, multilevel exploratory factor analysis (EFA) was conducted using Mplus v8.6 (Muthén & Muthén, 2021). Eigenvalues supported a one-factor model at both within- and between- persons levels, with all items demonstrating significant factor loadings (within-persons betas = 0.59-0.71; between-persons betas = 0.69-0.94). Multilevel reliability estimates supported adequate internal consistency on both within- (omega = 0.73) and between- (omega = 0.96) persons levels.

**Momentary affect.** At the beginning of each EMA prompt, participants reported on their current positive and negative affect using a 0-100 sliding scale (Table 2). Momentary PA was assessed using the following affect variables from the Positive Affect and Negative Affect Schedule-Child Form (PANAS-C; Laurent et al., 1999): happy, excited, joyful, and interested. Similar affect items have been used in prior studies with adolescents (e.g., Flores et al., 2018; Silk et al., 2011; Webb et al., 2022). Four discrete negative emotions – sad, worried, stressed, mad – were also assessed and used to create a composite score of momentary NA. Similar NA items have been used in prior adolescent research (e.g., Kenny et al., 2016; Silk et al., 2011).

**Table 2:** Summary of EMA items used in this study

Variable Name	EMA Item/Prompt	Response Type/Options
Assessed in all surveys		
Consummatory social reward	Think about the interaction with other kids your age that made you feel the best since the last beep. What happened? Check any statements that describe what you were thinking or feeling during the interaction (check all that apply):	Check-box format including 8 social reward statements ultimately summed: 1. I felt like someone was being friendly to me 2. I felt close to someone 3. I felt comfortable with someone 4. I felt like part of a group 5. I felt accepted or well-liked by someone 6. I felt like someone approved of or admired something I did 7. I had a lot of fun with someone 8. I got a compliment or praise from someone
Momentary PA	Please rate how you were feeling just before the phone beeped: Happy Excited Joyful Interested	All positive emotions rated on a sliding scale (0-100)
Momentary NA	Please rate how you were feeling just before the phone beeped: Sad Worried Stressed Mad	All negative emotions rated on a sliding scale (0-100)
Assessed in first (morning) survey only:		
Anticipatory social reward	What are you most looking forward to today?	0-100 sliding scale; also assessed with whom the event would be occurring

### Data Analytic Plan

#### Primary Analyses

The analytic plan and hypotheses were pre-registered following data collection but prior to data analyses: https://osf.io/4hx6w/?view_only=93aaa3d11876430faa5101074a5b3d96. Hypotheses were tested using a multilevel structural equation modeling (MSEM) approach implemented in Mplus v8.6 (Muthén & Muthén, 2021). MSEM represents a more flexible alternative to traditional multilevel modeling appropriate for the analysis of nested data (Preacher et al., 2011). Of particular relevance to the present work, MSEM decomposes variance into within- and between- person components in a model-driven manner using a latent variable approach and accommodates complex analyses of within- and between- person directional effects. Full-information maximum likelihood (FIML) estimation was used to account for missing data.

To evaluate associations between morning anticipatory social reward and same-day PA, we first created a daily PA score by taking the mean of each individual’s PA ratings across a given day. We then conducted an MSEM in which daily PA scores were regressed on morning anticipatory social reward ratings on both within- and between- person levels. To evaluate associations between consummatory social reward and momentary PA, we conducted an MSEM in which youth momentary PA ratings at the time of each survey were regressed on their rating of consummatory social reward in response to their self-identified best event since the last assessment at both the within- and between- persons level. Time since the event occurred was included as a covariate at the within-person level.

Finally, to evaluate associations between consummatory social reward on day t and anticipatory social reward and daily PA on day t + 1, we first calculated a day t consummatory social reward mean by averaging each individual’s consummatory social reward ratings across surveys within a given day.^3^ Then, we conducted a series of MSEMs in which next-day anticipatory social reward ratings and mean daily PA mean scores, respectively, were regressed on previous day mean consummatory social reward on within- and between- persons levels.

For all analyses, within-persons effects can be interpreted as the extent to which deviations from an individual’s own mean in one variable is associated with deviations from that individual’s own mean in another variable. Between-persons effects can be interpreted as the extent to which individual differences in one variable associate with individual differences in another variable.

### Sensitivity Analyses

To examine the extent to which patterns of findings were specific to PA relative to NA, additional analyses were conducted testing associations between anticipatory and consummatory reward and youth NA. Sensitivity analyses were conducted using an MSEM approach, analogous to the approach described above. These analyses were not preregistered.^4^

## Results

### Preliminary Analyses

Descriptive statistics characterizing primary variables of interest are reported in Table 3. For descriptive analyses, all variables were aggregated at the person-level by averaging across assessments. Within- and between- persons correlations characterizing key variables are also reported in Table 3. Anticipatory social reward was positively associated with momentary PA on both the within- (*r* = .21) and between- persons (*r* = .56) levels. Intraclass correlations indicated moderate between-persons stability in PA and reward across assessments.

Exploratory (not pre-registered) preliminary analyses examining associations between demographic variables (i.e., age, race, family income) revealed that age was positively associated with rate of endorsing anticipation of a peer event (*r* = .20, *p* = .029), as well as rate of endorsing having experienced a best event with peers (*r* =.30, *p* = .001). However, age was not correlated with the average intensity of youth’s anticipation of events with peers (*r*= -.04, *p* = .667) or their social reward in response to peer events (*r* = .07, *p* = .467). No other associations between demographic characteristics and primary variables of interest were found (all *p*s > 0.05).

**Table 3:** Descriptive statistics and bivariable correlations between key variables of interest

Descriptive Statistics	Mean	SD	ICC
1. Anticipatory reward	75.15	16.96	0.47
2. Social reward	2.06	1.40	0.36
3. Momentary PA	51.43	23.13	0.60
***Correlations***			
**Between-persons**			
	1	2	3
1. Anticipatory reward			
2. Social reward	0.11		
3. Momentary PA	**0.56**	0.11	
**Within-persons**			
1. Anticipatory reward			
2. Social reward	0.04		
3. Momentary PA	**0.21**	0.06	

*Note.* PA = positive affect; SD = standard deviation; ICC = intraclass correlation. For the purposes of descriptive analyses, all variables have been aggregated at the person-level via mean-scoring prior to calculation of grand-means and standard deviations. Correlations calculated using the ‘misty’ package (Yanagida, 2024) in R (R Core Team, 2022). Bolded values are statistically significant at *p* <.05

### Associations Between Anticipatory Social Reward and Daily PA

In morning surveys, 54.0% of EMA responses indicated anticipation of a social event with peers. Within-participants, the mean rate of endorsing anticipation of a peer event was 54.25% (SD = 22.14%). Only one participant, who completed 87% of surveys, reported never anticipating an event with peers.

Complete results of MSEM analyses are reported in Table 4. Intensity of youth anticipation of a peer event was positively associated with PA over the course of the day at both the within- (*b* = 0.19, *p* <.001) and between- persons (*b* = 1.04, *p* <.001) levels. At the within-person level, on days that youth experienced higher anticipatory social reward relative to their own average, they experienced higher PA relative to their own average for the rest of the day. At the between-person level, youth higher in anticipatory social reward relative to other youth experienced higher daily PA.

**Table 4:** Results of Multilevel Structural equation models (MSEMs) evaluating associations between Daily Life Social reward and positive affect

	b	SE (b)	*p*
*Model 1: Associations between Anticipatory Social Reward and Daily PA*			
***Within-Person*** **s**			
Anticipatory social reward → Daily PA	0.19	0.03	< 0.001
**Residual Variance**			
Daily PA	148.66	7.68	< 0.001
***Between-Persons***			
Anticipatory social reward → Daily PA	1.04	0.12	< 0.001
**Intercept**			
Daily PA	-28.32	9.31	0.002
**Residual Variance**			
Daily PA	283.73	45.79	< 0.001
*Model 2: Associations between Consummatory Social Reward and Momentary PA*			
***Within-Person*** **s**			
Consummatory social reward → Momentary PA	1.07	0.18	< 0.001
Time since the event	-1.08	0.13	< 0.001
**Residual Variance**			
Momentary PA	326.89	8.43	< 0.001
***Between-Persons***			
Consummatory social reward → Momentary PA	3.16	1.59	0.047
**Intercept**			
Momentary PA	49.93	3.93	< 0.001
**Residual Variance**			
Momentary PA	477.34	64.58	< 0.001
*Model 3: Associations between Consummatory Social Reward and Next Day Anticipation*			
***Within-Person*** **s**			
Consummatory social reward → Next day anticipation	− 0.24	0.54	0.664
**Residual Variance**			
Next day anticipation	407.51	22.01	< 0.001
***Between-Persons***			
Consummatory social reward → Next day anticipation	2.72	1.44	0.059
**Intercept**			
Next day anticipation	68.77	3.43	< 0.001
**Residual Variance**			
Next day anticipation	229.99	41.50	< 0.001
*Model 4: Associations between Consummatory Social Reward and Next Day PA*			
***Within-Person*** **s**			
Consummatory social reward → Next day PA	0.12	0.34	0.718
**Residual Variance**			
Next day PA	180.02	9.19	< 0.001
***Between-Persons***			
Consummatory social reward → Next day PA	3.17	1.91	0.096
**Intercept**			
Next day PA	43.99	4.52	< 0.001
**Residual Variance**			
Next day PA	523.84	41.50	< 0.001

*Note.* b = unstandardized effect size; PA = positive affect

### Associations Between Consummatory Social Reward and Momentary PA

Consummatory social reward was positively associated with subsequent PA at the within-person level (*b* = 1.07, *p* < 001): Increases in social reward during a recent positive peer event predicted higher than average momentary PA at the assessment most closely following the peer event, controlling for the time since the event occurred. Between-person associations between consummatory social reward and PA were also found (*b* = 3.16, *p* = .047; see Table 4); youth higher in consummatory social reward relative to other youth experienced higher momentary PA.

### Associations Between Consummatory Social Reward and Next Day Anticipatory Social Reward and PA

Consummatory social reward on day t was not significantly associated with next-day anticipatory reward^5^ at either the within- (*b*= -0.24, *p* = .664) or between- persons (*b* = 2.72, *p* = .059) levels. Associations between consummatory social reward at day t and next-day PA were also not significant (within-persons: *b* = 0.12, *p* = .718; between-persons: *b* = 3.17, *p* = .096; see Table 4).

### Sensitivity Analyses

Anticipatory 6 social reward in the morning was not related to youth NA over the course of day at either the within- or between- person levels (*p*s > 0.05). Consummatory social reward was similarly unrelated to youth subsequent NA at either the within- or between- persons levels (*p*s > 0.05; see Table 5 for full results).

**Table 5:** Results of sensitivity analyses

	b	SE (b)	*p*
*Model 1: Associations between Anticipatory Social Reward and Daily NA*			
***Within-Person*** **s**			
Anticipatory social reward → Daily NA	− 0.001	0.02	0.949
**Residual Variance**			
Daily NA	51.94	2.68	< 0.001
***Between-Persons***			
Anticipatory social reward → Daily NA	− 0.05	0.07	0.450
**Intercept**			
Daily NA	12.85	5.14	0.012
**Residual Variance**			
Daily NA	95.83	14.02	< 0.001
*Model 2: Associations between Consummatory Social Reward and Momentary NA*			
***Within-Person*** **s**			
Consummatory social reward → Momentary NA	− 0.07	0.11	0.549
Time since the event	0.19	0.08	0.015
**Residual Variance**			
Momentary NA	124.15	3.20	< 0.001
***Between-Persons***			
Consummatory social reward → Momentary NA	0.20	0.72	0.780
**Intercept**			
Momentary NA	7.67	1.80	< 0.001
**Residual Variance**			
Momentary NA	95.00	13.18	< 0.001

*Note.* b = unstandardized effect size; NA = negative affect

## Discussion

In this study, intensive longitudinal methods were used to characterize associations between real-world anticipatory social reward, consummatory social reward, and momentary positive affect (PA) in adolescent girls. Anticipating and perceiving high levels of social reward in daily interpersonal interactions, particularly with peers, may play a crucial role in social development and belongingness for adolescents, who are highly sensitive to peer evaluation and feedback and tasked with joining peer groups (Blakemore & Mills, 2014). Given the salience and centrality of social relationships for adolescents, enhancing social reward is often a key focus of positive youth development and intervention for psychopathology. Adolescent girls may be particularly likely to benefit from enhancing social reward. By testing baseline associations between real-world social reward processes and momentary PA in adolescent girls at temperamental risk for social anxiety and depression, this study will inform future efforts that aim to increase social reward in girls to improve mental health and well-being.

As hypothesized, girls reported higher PA on days that they reported higher anticipatory social reward in the morning. Additionally, girls reported higher PA following a more socially rewarding interaction with a peer. Exploratory analyses (not pre-registered) showed that associations between anticipatory or consummatory social reward and momentary affect were specific to PA; neither anticipatory nor consummatory social reward were associated with momentary NA. This finding may be particularly important to replicate in future research, as it speaks to specificity that may have high clinical importance (i.e., interventions targeting social interest and reward may be particularly important for individuals with blunted PA).

Interestingly, no associations between day-level consummatory social reward and next-day PA or anticipatory social reward were found, which could suggest that perceiving more social reward in peer interactions may not have long-lasting impacts on PA or how much girls look forward to socializing the next day. This finding is consistent with prior work showing that PA predicts engagement in healthy behaviors within the same day but not across days (Aurora et al., 2022). If replicated, these findings could suggest that interventions aimed at increasing youths’ sense of reward in peer interactions may not have substantial long-term impacts on positive emotional health or social interest. However, high consummatory social reward could impact other positive emotions, such as belongingness, that were not assessed. There might also be important effects of timescale here. We examined how consummatory social reward at day t predicts anticipatory social reward at day t + 1 (which was only assessed at each morning observation), but it is possible that effects unfold over shorter or more protracted timescales. Further, experiences of consummatory reward may have more fleeting effects on anticipatory social reward or accumulate over time to contribute to longer-term increases in anticipatory social reward. It is also important to note that the present sample was not a clinical sample; replicating findings in other, more diverse samples, particularly clinical samples, will help clarify the clinical implications of these findings.

Some additional limitations are important to note here. First, unexpected data loss precluded our ability to examine associations between anticipation of a positive peer event and enjoyment of that event reported at the end of the day, as there were very few positive peer social events that were identified in the morning and reported on again at the end of the day. This is mainly because participants were not asked to report specifically on social events each morning; participants could report on anything they were looking forward to that day and only some events were both social and peer-related in nature. Examining how anticipation of an event contributes to actual enjoyment of that event, and how associations between social reward anticipation and consummation are associated with PA, remains an interesting area for future work to explore. Additionally, the social reward EMA items were created for this study and have not been published previously; thus, data on psychometric properties of these variables are limited. Nevertheless, we demonstrate that the EMA measure of consummatory social reward is reliable. In addition, we have previously shown that a similar EMA measure of social threat, which indexes how socially threatened youth feel in negative peer interactions, is reliable and valid (Sequeira et al., 2021). Further, previous studies have shown that single-item EMA measures (e.g., our anticipatory social reward item) show strong concurrent and predictive validity (e.g., Song et al., 2023).

Exploratory (pre-registered) analyses did not identify differences in associations between real-world social reward processes and PA by temperament status. However, it is important to note that this sample was not recruited with the goal of testing group differences, but rather to increase variability in social anxiety symptoms, thus helping to explain unequal temperament group sizes. Further, as this study recruited only girls, it will be important to examine relationships between social interest, reward, and PA in adolescent boys in future work. As girls are more sensitive to social evaluation (Rudolph & Conley, 2005) and at higher risk for psychological disorders associated with aberrant PA (e.g., depression, social anxiety) relative to boys (Merikangas et al., 2010), it is possible that the associations between social reward processes and PA may be stronger in girls relative to boys, but we were unable to test this. Examining sex differences directly in future studies would provide valuable insight. Additional factors not assessed in this study may also influence associations between anticipatory and consummatory social reward and PA. For example, youth experiencing more family conflict might benefit more from perceiving more rewarding peer interactions.

As noted in the introduction, associations between social reward processes and PA are likely reciprocal, such that experiencing higher PA may also lead one to anticipate more socially rewarding interactions and extract more social reward in social interactions. These reciprocal effects are important to test in future research designed to test such time-lagged events. In the present study, we pre-registered only hypotheses about the effects of social reward on PA (and consummatory social reward on anticipatory social reward). We chose not to test effects of PA on social reward because we lacked proximal measures of PA and subsequent social reward. Moreover, PA was not assessed in the morning prior to the assessment of anticipatory social reward. Additionally, the time between EMA assessments varied by person and a participant’s best peer interaction reported at each assessment could have occurred at any point since the last assessment; with the adjustments for time that would be needed, findings that might arise from these models could be challenging to interpret.

Despite these limitations, the present study helps us understand how changes in real-world anticipatory and consummatory social reward are associated with PA in early adolescent girls. These findings may be particularly relevant for researchers and clinicians designing and implementing psychological interventions for girls low in PA. Findings are interesting to consider in the context of clinical studies in adults with severely low PA, which show that increasing reward anticipation-motivation and responses to reward attainment in treatment is associated with increases in PA (Craske et al., 2023). Taken together, results from this latter study and the current study suggest that prevention and intervention efforts to increase social approach motivation (e.g., through behavioral activation) and reward extracted from social interactions (e.g., through savoring) may have positive impacts on PA for individuals with, or at risk for, low PA.

## Footnotes

1 Given the nature of the sample, several hypotheses related to temperament status were also pre-registered. Specifically, we hypothesized that relative to girls low-moderate in shy/fearful temperament, girls high in shy/fearful temperament would report looking forward to a positive event with peers more (on average), which could be related to heightened approach motivation in this population (Barker et al., 2019; Helfinstein et al., 2012). Additionally, potential differences in consummatory social reward or pleasure and momentary PA between girls high and low-moderate in shy/fearful temperament were explored. Temperament status was also explored as a potential moderator on the associations between consummatory social reward or pleasure and momentary PA. Results indicated that high and low temperamental risk groups did not significantly differ on key variables or associations of interest; no significant group differences were found. Interested readers are encouraged to contact study authors for further details.

2 We originally pre-registered a hypothesis that looking forward to a peer social event more would be associated with enjoying that peer event more, though we were unable to test this hypothesis due to lack of available data.

3 Pre-registered materials initially specified that consummatory social reward in this analysis would be represented using youth’s rating of the extent to which they enjoyed the peer event that they had previously reported to be their most highly anticipated event of the day (“Did the thing you were looking forward to happen?” [if yes]: “How much did you enjoy it?“) This item was delivered at the final assessment of each day. If the event occurred, participants rated how much they enjoyed it on a 0-100 sliding scale. Upon reviewing the data, however, it was noted that use of this item would result in significantly reduced power due to restricted sample size. Thus, the decision was made not to use this social pleasure variable and instead to represent consummatory social reward in this analysis as an aggregated score of youth’s social reward in recent peer interactions throughout the day (described in Measures). Although this approach is limited in its ability to yield unique insights into associations between anticipation and enjoyment of the same event, we feel that this approach adequately addresses primary aims of understanding associations between anticipatory and consummatory social reward more broadly and enhances reliability and validity through the use of multiple indicators (i.e., repeated assessments of social pleasure over a given day).

4 The present study pre-registration also specified that sensitivity analyses would be conducted excluding youth who completed fewer than three surveys. Upon review of the data, however, it was noted that all participants completed more than three surveys. Thus, these pre-registered analyses were not conducted.

5 For the purposes of these analyses, we considered anticipatory reward for all social events (not limited to anticipated peer events) in the interest of power.

6 Multigroup Models were also conducted to evaluate potential differences in patterns of effects across temperament groups (i.e., high versus low temperamental risk). These analyses did not detect meaningful group differences in effects, although it must be noted that due to limitations related to sample size, the present work was likely underpowered to detect small to moderate differences across groups. For this reason, results are not reported in the present manuscript; however, interested readers are encouraged to contact study authors for further details.
